# Chronic Psychosocial Stress and Negative Feedback Inhibition: Enhanced Hippocampal Glucocorticoid Signaling despite Lower Cytoplasmic GR Expression

**DOI:** 10.1371/journal.pone.0153164

**Published:** 2016-04-08

**Authors:** Andrea M. Füchsl, Stefan O. Reber

**Affiliations:** Department of Behavioural and Molecular Neurobiology, University of Regensburg, 93053, Regensburg, Germany; Radboud University Medical Centre, NETHERLANDS

## Abstract

Chronic subordinate colony housing (CSC), a pre-clinically validated mouse model for chronic psychosocial stress, results in increased basal and acute stress-induced plasma adrenocorticotropic hormone (ACTH) levels. We assessed CSC effects on hippocampal glucocorticoid (GC) receptor (GR), mineralocorticoid receptor (MR), and FK506 binding protein (FKBP51) expression, acute heterotypic stressor-induced GR translocation, as well as GC effects on gene expression and cell viability in isolated hippocampal cells. CSC mice showed decreased GR mRNA and cytoplasmic protein levels compared with single-housed control (SHC) mice. Basal and acute stress-induced nuclear GR protein expression were comparable between CSC and SHC mice, as were MR and FKBP51 mRNA and/or cytoplasmic protein levels. *In vitro* the effect of corticosterone (CORT) on hippocampal cell viability and gene transcription was more pronounced in CSC versus SHC mice. In summary, CSC mice show an, if at all, increased hippocampal GC signaling capacity despite lower cytoplasmic GR protein expression, making negative feedback deficits in the hippocampus unlikely to contribute to the increased ACTH drive following CSC.

## Introduction

The homeostasis of an individual is constantly challenged by acute internal or external disturbances, also called stressors. A proper stress response, i.e. a fast and timely limited activation of both the hypothalamo-pituitary-adrenal (HPA) axis as well as the sympathetic nervous system, in turn facilitates the restoration of homeostasis and, thus, promotes survival (for review see [1, 2]). However, chronic or repeated stressor exposure, at least in part via altering the negative feedback system, often leads to a dysregulation of normal HPA axis functionality and a prolonged activation of the stress axis also in response to acute and non-harmful challenges (for review see [1]), [3]. Interestingly, alterations in the negative feedback response, due for instance to changes in glucocorticoid (GC) signaling, are discussed to promote the development of stress-related psychiatric disorders, like major depression, posttraumatic stress disorder and anxiety disorders (for review see [1, 4, 5]).

Glucocorticoids (corticosterone (CORT) in rats and mice, cortisol in humans) signal through two different cytoplasmic receptors, the mineralocorticoid receptor (MR) and the glucocorticoid receptor (GR). The MR binds GC with high affinity and, therefore, is occupied already at low GC levels. The GR, in contrast, has a ten-fold lower affinity and, thus, is only occupied under conditions of high GC levels, i.e. at the circadian peak and during stressor exposure (for review see [6]), [7]. While the GR is widely distributed throughout the brain, the MR is mainly localized in limbic brain structures, like the hippocampus, lateral septum, amygdala, hypothalamus, and medial prefrontal cortex (PFC) [6, 8].

MR and GR are ligand dependent transcription factors, whose inactive form is associated with a chaperone complex in the cytoplasm (for review see [9]), [10]. One of these chaperones is the immunophilin FK506-binding protein 51 (FKBP51). Given that a GC-induced increase in FKBP51 expression in turn reduces ligand binding and translocation of the GR, this chaperone is hypothesized to be involved in the termination of GR signaling via an ultra-short feedback loop (for review see [11]). In the nucleus MR and GR can either bind as homo- or heterodimer to GC-responsive elements (GRE) in the promoter region of target genes (transactivation) or interact with other transcription factors (transrepression) (for review see [12]). Meanwhile, it is further commonly accepted that besides the classical cytoplasmic corticosteroid receptors there are also membrane-associated ones mediating the rapid, non-genomic effects of GC (for review see [13]).

HPA axis reactivity to stress is mainly controlled by the pituitary [14], the hippocampus, the paraventricular nucleus (PVN) of the hypothalamus [15, 16] and the medial PFC [17]. The hippocampus mainly influences the PVN via innervating the posterior bed nucleus of the stria terminalis, which relays the information to the PVN (for review see [18]), [19]. Given the marked presence of MR and GR in the hippocampus [6], this brain region turned out to be extremely sensitive to the deleterious consequences of a prolonged elevation in plasma GC [20, 21], amongst others promoting morphological changes including for instance hippocampal volume loss [22] and dendritic atrophy (for review see [23]).

Chronic subordinate colony housing (CSC, 19 days), an established and pre-clinically validated model for chronic psychosocial stress in male mice, has repeatedly been shown to induce alterations in HPA axis functionality as well as somatic and affective pathologies [24, 25], (for review see [26]). CSC mice for instance are characterized by elevated basal as well as acute stress-induced plasma adrenocorticotropic hormone (ACTH) concentrations compared to single-housed control (SHC) mice [27]. Although a decreased cytoplasmic pituitary GR expression at the first glance suggested a compromised negative feedback inhibition to mediate pituitary hyperactivity, an unaffected or even increased dexamethasone suppression during acute stressor exposure clearly argued against this hypothesis, at least at the level of the pituitary [27]. However, it remains to be shown that also further upstream brain sites involved in the negative feedback inhibition of the HPA axis, like the hippocampus, are not compromised by CSC exposure, especially given that GC resistance was found in both splenocytes [24] and T helper 2 cells from peripheral lymph nodes [28] of CSC compared with SHC mice.

Therefore, in the present study we assessed i) hippocampus weight and hippocampal cell number, ii) hippocampal MR, GR and FKBP51 mRNA and/ or cytoplasmic protein expression, iii) GR nuclear protein expression under basal and acute stress conditions (elevated platform, EPF, 5 min), iv) *in vitro* ability of CORT to induce hnRNA expression of the GC-responsive gene Period 1 (Per1) [29], and v) cell viability of isolated hippocampal cells in response to 24 h of CORT stimulation *in vitro*. Adrenal weight, plasma CORT and ACTH concentrations were assessed to confirm reliability and reproducibility of the CSC paradigm.

## Material and Methods

### Animals

Male C57BL/6 mice (Charles River, Sulzfeld, Germany) weighing 19–22 g (experimental mice) were individually housed in standard polycarbonate mouse cages (16 x 22 x 14 cm) for one week before the CSC paradigm started. The male offspring (weighing 30–35 g) of high anxiety-related behaviour female mice (kindly provided by Prof. Dr. R. Landgraf, Max Planck Institute of Psychiatry in Munich) and C57BL/6 male mice (Charles River, Sulzfeld, Germany) were used as dominant animals. All mice were kept under standard laboratory conditions (12 h light/ 12 h dark cycle, lights on at 0600 h, 22°C, 60% humidity) and had free access to tap water and standard mouse diet. All experimental protocols were approved by the Committee on Animal Health and Care of the local government, and conformed to international guidelines on the ethical use of animals. All efforts were made to minimize the number of animals used and their suffering.

### Experimental procedures

All experimental mice were either chronically stressed by 19-day exposure to the chronic subordinate colony housing (CSC) paradigm or single-housed for control (SHC). On day 20 of CSC, 4 sets of SHC and CSC mice were decapitated between 0800 and 1000 h and the hippocampi were removed for assessment of mRNA levels of MR and GR (1^st^ set; SHC: n = 13; CSC: n = 19), cytoplasmic protein levels of MR, GR, and FKBP51 and basal nuclear protein levels of GR (2^nd^ set; SHC: n = 8; CSC: n = 6), *in vitro* CORT-induced Per1 hnRNA expression in isolated hippocampal cells (3^rd^ set; SHC: n = 14; CSC: n = 14), and *in vitro* cell viability of isolated hippocampal cells in response to CORT stimulation (4^th^ set; SHC: n = 23; CSC: n = 24). To determine acute stress-induced nuclear translocation of the GR, one additional set of SHC and CSC mice (5^th^ set; SHC: n = 6; CSC: n = 7) was exposed to the elevated platform (EPF, 5 min) on day 20 of CSC and killed 10 min following termination of the acute stressor for assessment of nuclear GR protein levels in the hippocampus. Absolute hippocampus weight was assessed in set 1 and 2 of SHC and CSC mice and hippocampal cell number was assessed in set 3 and 4 of SHC and CSC mice. Hippocampi from all animals in set 1, and from some animals in set 2 and 4, stem from mice which served as experimental CSC or SHC mice in studies published recently [25, 30], ensuring the reliability/ reproducibility of the CSC paradigm. To verify reliability/ reproducibility of the CSC model also for the remaining mice (SHC: n = 36; CSC: n = 42) in set 2, 3, and 4 of the current study, adrenals were taken and adrenal weight was provided. Furthermore, basal plasma CORT (SHC: n = 47; CSC: n = 53) and ACTH (SHC and CSC: n = 5) concentrations were assessed.

### Chronic subordinate colony housing (CSC)

The chronic subordinate colony housing (CSC) paradigm was conducted as described previously [24, 25, 30–32]. Briefly, four experimental CSC mice were housed together with a dominant male mouse for 19 consecutive days, in order to induce a chronic stressful situation. Before the CSC procedure, the future dominant males were tested for their aggressive behaviour. To confirm the intended dominant/ subordinate hierarchy within each colony, mice were always videotaped during the first 1 h after putting them together on days 1, 8, and 15. Males that started to injure their opponents by harmful bites were not used. To avoid habituation, each dominant male was replaced by a novel dominant male at days 8 and 15 of the CSC procedure. SHC mice remained undisturbed in their home cages except for change of bedding once a week. In a previous study we convincingly demonstrated that single housing is the adequate control group for the CSC paradigm, as group housing itself was shown to be stressful and to affect parameters assessed routinely in studies employing the CSC paradigm [33].

### Elevated platform (EPF) exposure

In the morning of day 20 between 0800 and 1000 h, SHC and CSC mice were exposed to an EPF for 5 min [25]. Each mouse was individually placed in the center of the EPF (diameter 18 cm; elevation 75 cm; 160 lux). 10 min following termination of the EPF exposure, SHC and CSC mice were decapitated under CO_2_ anaesthesia and the left and right hippocampus of each mouse was removed and stored at -80°C. The EPF was cleaned thoroughly before each trial.

### Determination of absolute adrenal weight

After decapitation under CO_2_ anaesthesia on day 20, the left and right adrenal of each mouse was removed, pruned of fat, pooled, and weighed.

### Determination of plasma CORT and ACTH

After decapitation under CO_2_ anaesthesia on day 20, trunk blood was collected in chilled EDTA-coated tubes and kept on ice until all animals have been killed. Afterwards blood samples were centrifuged (5000 g, 10 min, 4°C) and plasma was stored at -20°C until assayed. Plasma samples were analyzed using commercially available ELISA kit for CORT (analytical sensitivity < 1.631 nmol/l, intra-assay and inter-assay coefficients of variation ≤ 6.35%, IBL International, Hamburg, Germany) and ACTH (analytical sensitivity 0.22 pg/ml, intra-assay and inter-assay coefficients of variation ≤ 7.1%, IBL International, Hamburg, Germany).

### Determination of hippocampus weight

After decapitation under CO_2_ anaesthesia on day 20, the left and right hippocampus of each mouse was removed, pooled, and weighed. Hippocampi were either stored in RNAlater^®^ Stabilization Solution (Applied Biosystems, Forster City, CA, USA) for determination of MR and GR mRNA expression or frozen in liquid nitrogen and stored at -80°C for determination of MR, GR and FKBP51 protein expression. Due to methodological problems four SHC and one CSC mouse had to be excluded from hippocampus weight analysis.

### Quantitative real-time polymerase chain reaction (qPCR) using TaqMan technology

Total RNA was prepared from hippocampal tissue or hippocampal cells (for Per1 hnRNA) using the RNeasy Mini Kit (Qiagen, Hilden, Germany) and reversely-transcribed into first-strand cDNA (Reverse Transcription System, Promega, Mannheim, Germany) as described previously [25, 31, 34].

Expression levels of GR and MR mRNA in the hippocampus were quantified by TaqMan-qPCR (ABI PRISM 7900HT Sequence Detection System, Applied Biosystems, Foster City, CA, USA). Primers used were as follows: GR forward: CGGGACCACCTCCCAAA, GR reverse: CCCCATAATGGCATCCCGAA, GR probe: TCTGCCTGGTGTGCTCCGATGAAG, MR forward: GGACCAAATTACCCTCATCCA, MR reverse: GTATGTTTGTACGATCTCCAACTCAAG, MR probe: ATTCTTGGATGTG TCTATCATC. The probes were labelled 5`with 6-carboxyfluorescein (FAM) and 3`with 6-carboxytetramethylrhodamine (TAMRA). TaqMan-qPCR was performed using 1 μl cDNA, 1 μl forward and reverse primer each (18 μM), 1 μl probe (5 μM), 10 μl TaqMan Mastermix (Applied Biosystems, Foster City, CA, USA), 1 μl glyceraldehyde-3-phosphate-dehydrogenase (GAPDH)-Mix (served as reference; Applied Biosystems, Foster City, CA, USA) and made up to the final volume of 20 μl with sterile H_2_O. Cycling was performed as follows: 50°C for 2 min, 95°C for 10 min followed by 40 repeats: 95°C for 15 s and 60°C for 1 min.

Expression levels of Per1 hnRNA in isolated hippocampal cells were quantified by TaqMan-qPCR (ABI 7500 Fast Real-Time PCR System; Applied Biosystems, Foster City, CA, USA). Primers used were as follows: Per1 forward: 5’ GTGCGCACGTAAGGGAACTG 3’, Per1 reverse: 5’ CCCATGCCATGTCCATACC 3’, Per1 probe: 5’ TCTCCACGCTGGTGTT 3’. The probes were labelled 5`with FAM and 3`with TAMRA. TaqMan-qPCR was performed using 2 μl cDNA, 0.3 μl forward and reverse primer each (150 nM), 0.35 μl probe (350 nM), 10 μl 2 x Brilliant III Ultra-Fast QPCR Master Mix (Agilent Technologies, Santa Clara, CA, USA), 0.6 μl GAPDH-Mix, and made up to the final volume of 20 μl with sterile H_2_O. Cycling was performed as follows: 95°C for 3 min followed by 40 repeats: 95°C for 15s and 60°C for 30s.

Expression value normalized to GAPDH mRNA expression was quantified for each mouse and averaged per group.

### Cytoplasmic and nuclear protein extraction

For analysis of cytoplasmic GR, MR and FKBP51 and of nuclear GR expression frozen hippocampi protein extraction was performed according to the method of Vallone et al. [35–37]. Hippocampi were homogenized in S1 buffer (10 mM HEPES (pH 7.9), 10 mM KCl, 1.5 mM MgCl_2_, 0.1 mM EDTA (pH 8)) supplemented with 0.5 mM dithiothreitol, 0.2 mM Na-orthovanadate, 2 mM NaF and complete mini protease inhibitor (Roche Diagnostics GmbH, Mannheim, Germany) and then centrifuged for 10 min at 7000 rpm at 4°C. The supernatant was used for cytoplasmic extraction and the pellet was used for extraction of nuclear proteins. For the cytoplasmic fraction, the supernatant was centrifuged again for 30 min at 13.000 rpm at 4°C and the final supernatant was stored at -80°C. The pellet was resuspended in an appropriate volume of S1 buffer and passed through a 25 gauge needle. The suspension was centrifuged for 10 min at 5000 rpm at 4°C and the pellet was washed two times with S1 buffer. Finally, the nuclear proteins were extracted in 1.2 pellet volume of cold S2 buffer (10 mM HEPES (pH 7.9), 400 mM NaCl, 1.5 mM MgCl_2_, 0.1 mM EDTA (pH 8), 5% glycerol, supplemented as described above), incubated for 1h on ice and centrifuged for 30 min at 13.000 rpm at 4°C. The supernatant of the nuclear fraction was stored at -80°C. Total protein concentrations were determined using a commercial kit (Bicinchoninic Acid Protein Assay Kit, Thermo Scientific, Rockford, USA).

### Western Blotting

Western Blotting was performed as described previously [25, 27, 30] using equal amounts of protein lysates (20 μg) and antibodies for rabbit anti-mouse GR (1:200, Santa Cruz Biotechnology, Inc., Heidelberg, Germany), goat anti-mouse FKBP51 (1:500, Santa Cruz Biotechnology, Inc., Heidelberg, Germany) and rabbit anti-mouse MR (1:200, Santa Cruz Biotechnology, Inc., Heidelberg, Germany). After incubation with donkey anti-goat IgG horseradish peroxidase (HRP)-conjugated (1:6000 for FKBP51, Santa Cruz Biotechnology, Inc., Heidelberg, Germany) or HRP-conjugated whole goat anti-rabbit secondary antibody (1:5000 for GR, 1:2000 for MR, Cell Signaling Technology, New England Biolabs GmbH, Frankfurt am Main, Germany), antibody binding was visualized on Molecular Imager^®^ ChemiDoc^™^ XRS+ system (Bio-Rad Laboratories, München, Germany) using an Enhanced Chemiluminescent Western Blotting detection reagent (GE Healthcare, Freiburg, Germany).

Afterwards, each membrane was stripped using Re-Blot Plus Antibody Stripping Solution (Millipore GmbH, Schwalbach, Germany) and probed with primary rabbit anti-ß-Tubulin antibody (1:1000, Cell Signaling Technology, New England Biolabs GmbH, Frankfurt am Main, Germany) as loading control for cytoplasmic and with primary rabbit anti TATA binding protein (TBP, 1:200, Abcam plc, Cambridge, UK) as loading control for nuclear protein. Visualization and digitization was performed as described above. Semiquantitative densitometric analyses of the signals were performed using Image Lab^™^ Software (Bio-Rad Laboratories, München, Germany). GR (~ 86 kDa), FKBP51 (~ 51 kDa) and MR (~107 kDa) protein expression for each mouse was normalized to ß-Tubulin (~ 50 kDa, cytoplasmic fraction) or TBP (~ 38 kDa, nuclear fraction) expression and averaged per group. Owing to methodological problems, for MR two SHC mice and for nuclear GR two SHC and one CSC mice had to be excluded from the protein expression analysis.

### Hippocampal cell isolation

For the hippocampal cell stimulation, left and right hippocampi of each mouse were pooled. After dissection, hippocampi were collected in 1.8 ml ice-chilled Hanks’ balanced salt solution (HBSS, Invitrogen GmbH, Karlsruhe, Germany). Thereafter, 200 μl Trypsin (Sigma Aldrich, Deisenhofen, Germany) were added and the hippocampi were incubated for 20 min at 37°C/ 5% CO_2_. Next, 10 mg/ ml Deoxyribonuclease (Sigma Aldrich, Deisenhofen, Germany) was added for 30 s in order to break down DNA and to avoid clumping of tissue during the subsequent trituration. Afterwards the tissue was washed once with HBSS/ 10% fetal bovine serum (FBS, PAA Laboratories GmbH, Cölbe, Germany) to stop digestion and twice with HBSS. After discarding the supernatant, the tissue was homogenized with a plastic pasteur-pipette in DMEM (Invitrogen GmbH, Karlsruhe, Germany) and centrifuged at 1000 rpm for 10 min at 20°C. The supernatant was again discarded, the pellet was resuspended in DMEM and the number of viable cells was determined using a cell viability analyzer (Vi-Cell XR, Beckmann Coulter, Krefeld, Germany). Afterwards the cell suspension was again centrifuged at 1000 rpm for 10 min at 20°C and the pellet was either resuspended in DMEM/ 0.1% BSA for the determination of the cell viability after 24 h of CORT incubation or in DMEM without BSA for the measurement of CORT-induced Per1 hnRNA expression. As BSA is necessary for cell survival but also contains small amounts of GC, it was not added during the relatively short CORT incubation period applied for induction of Per1 hnRNA expression (30 min) but was added during 24 h stimulation applied for assessment of cell CORT effects on cell viability. All work was done under sterile conditions.

### Hippocampal gene expression in response to CORT treatment

For measurement of the CORT-induced expression of Per1 hnRNA, 7 x 10^4^ viable hippocampal cells per well were plated onto a Poly-L-Lysine (Sigma Aldrich, Deisenhofen, Germany) coated 96-well plate and incubated for 1 h at 37°C/ 5% CO_2_. Afterwards equal volumina of vehicle or CORT were added (final CORT concentrations of either 0.1 μM or 100 μM) for 30 min at 37°C/ 5% CO_2_. CORT was first dissolved in EtOH (60%) and then further diluted to the appropriate concentration with saline. Vehicle consisted of saline containing comparable amounts of EtOH as used in the respective CORT stimulation condition. Thereafter, the samples were centrifuged for 8 min at 800 rpm, the supernatant was discarded and the pellet was resuspended in RLT buffer (Qiagen, Hilden, Germany) containing 1% beta-mercaptoethanol. The cell solution was stored at -80°C until RNA extraction and qPCR were performed (see above).

### Hippocampal cell viability measurement in response to CORT treatment

2 x 10^4^ viable hippocampal cells per well were plated onto a Poly-L-Lysine (Sigma Aldrich, Deisenhofen, Germany) coated 96-well plate and incubated with different CORT concentrations (0.1 μM, 1 μM, 10 μM, 100 μM, 1000 μM) (Sigma Aldrich, Deisenhofen, Germany) or vehicle for 24 h at 37°C/ 5% CO_2_. CORT was first dissolved in Ethanol (EtOH, 60%) and then further diluted to the appropriate concentrations with saline. Vehicle consisted of saline containing comparable amounts of EtOH as used in the respective CORT stimulation condition. The cell viability of the hippocampal cells was determined 24 h later with a commercially available colorimetric assay (Cell Titer AQ_ueous_ One Solution Cell Proliferation Assay Promega, Mannheim, Germany), by measuring the absorbance at a wavelength of 485 nm by using an ELISA plate reader (BMG Labtech GmbH, Ortenberg, Germany). To account for differences in background activity, the absorbance of wells containing only medium for a given treatment was subtracted from the corresponding wells containing cells.

### Statistics

For statistical comparisons, the software package SPSS statistics (version 19.0) was used. Data of two experimental groups (SHC versus CSC) were analyzed using the parametric Student’s *t*-test. Nuclear GR protein expression in response to acute stressor exposure (EPF, 5 min; factor CSC and factor EPF), hippocampal cell viability and Per1 hnRNA expression in response to *in vitro* CORT stimulation (factor CSC and factor CORT) were compared using a two-way ANOVA. ANOVA was followed, when a significant main effect was found, by *post-hoc* analysis using Bonferroni pairwise comparison. Data represent the mean + SEM. The level of significance was set at *P* < 0.05.

## Results

### CSC increased absolute adrenal weight

**Fig 1:**
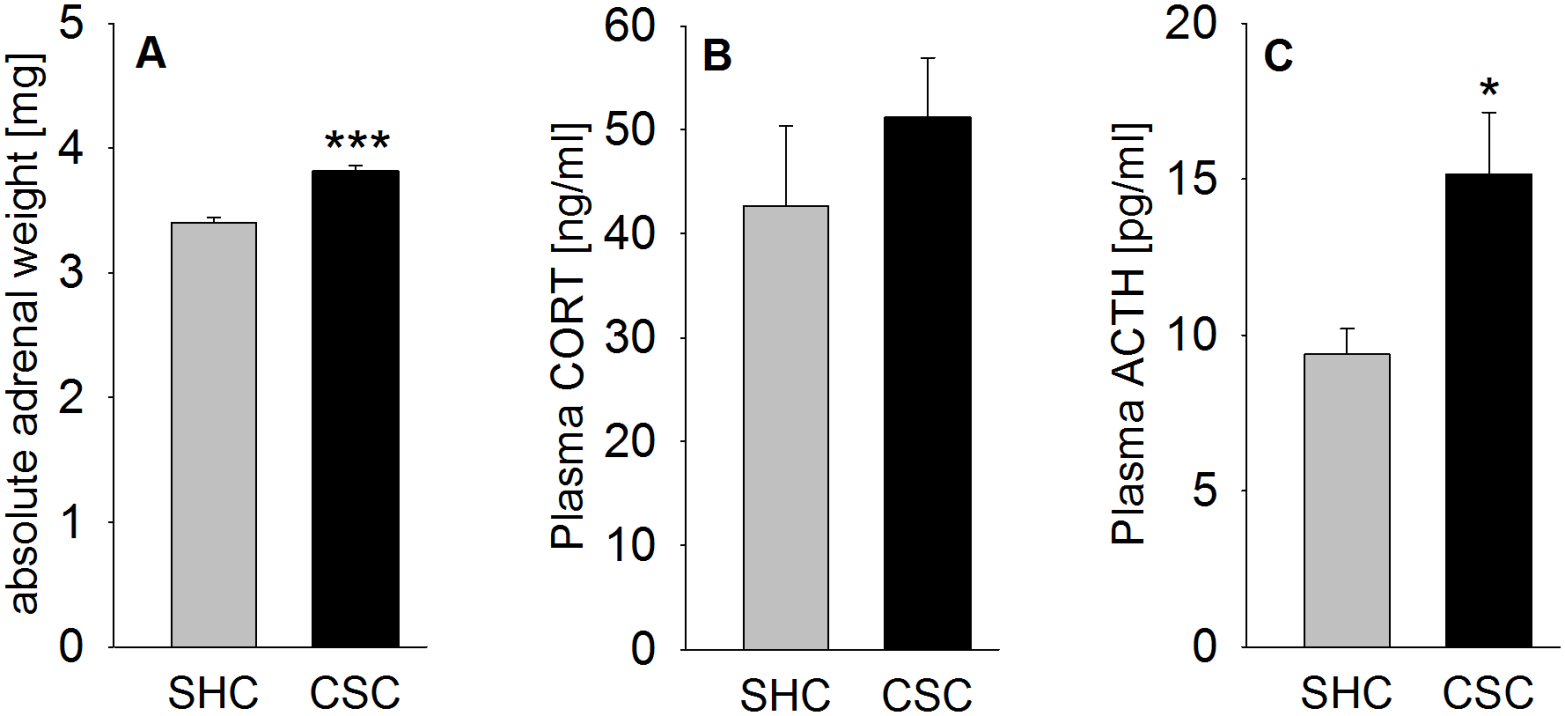
Effects of CSC on absolute adrenal weight and plasma CORT and ACTH concentrations. Following decapitation on day 20 of CSC between 0800 and 1000 h under basal conditions the left and right adrenals of SHC (n = 36) and CSC (n = 42) mice were removed, pruned of fat, pooled, and weighed. Depicted is the absolute weight [mg] of the left and right adrenal (sum of both, A). Furthermore, plasma CORT [ng/ml] (SHC: n = 47; CSC: n = 53; B) and ACTH [pg/ml] (SHC and CSC: n = 5; C) concentrations were determined in trunk blood following decapitation. Grey bars represent SHC, black bars CSC mice. Data represent the mean + SEM. * represent *P* < 0.05, *** represent *P* < 0.001 vs. respective SHC mice.

Statistical analysis revealed an increase in absolute adrenal weight (*P* < 0.001; Fig 1A) in CSC compared with SHC mice.

### CSC increased plasma ACTH but did not affect plasma CORT concentrations

Statistical analysis revealed an increase in plasma ACTH concentrations (*P* = 0.028; Fig 1C) but not in plasma CORT concentrations (Fig 1B) in CSC compared with SHC mice

### CSC did not affect absolute hippocampus weight and hippocampal cell number

**Fig 2:**
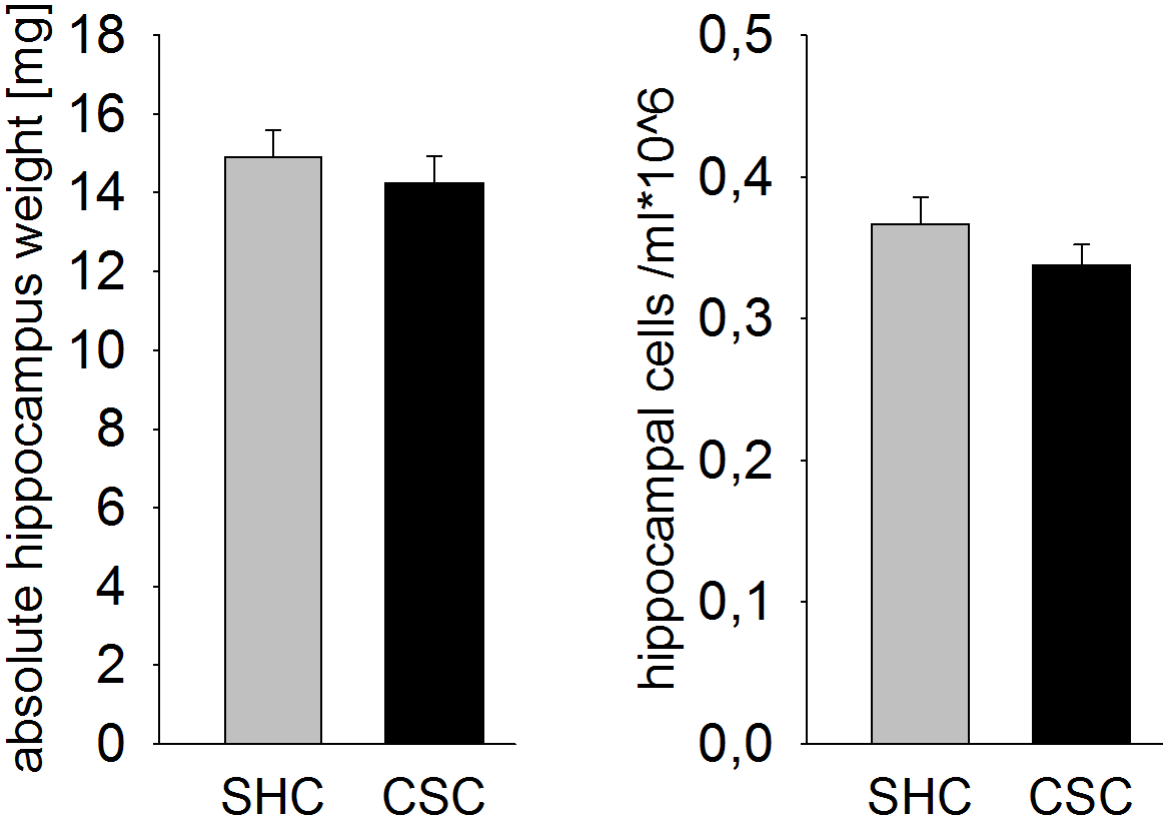
Effects of CSC on hippocampus weight and on hippocampal cell number. Following decapitation on day 20 of CSC the left and right hippocampus of SHC (n = 17) and CSC (n = 24) mice were removed, pooled and weighed. Depicted is the absolute weight [mg] of the left and right hippocampus (sum of both, A). Hippocampal cells of SHC (n = 37) and CSC (n = 38) mice were isolated and subsequently quantified (per ml) by means of a Cell Viability Analyser (B). Grey bars represent SHC, black bars CSC mice. Data represent the mean + SEM.

Statistical analysis revealed that CSC had no effect on the absolute hippocampus weight [mg] (Fig 2A) as well as on the number of isolated hippocampal cells (Fig 2B).

### CSC decreased GR mRNA and cytoplasmic protein expression and had no effect on MR mRNA and cytoplasmic protein as well as on basal and EPF-induced nuclear GR protein expression in the hippocampus

**Fig 3:**
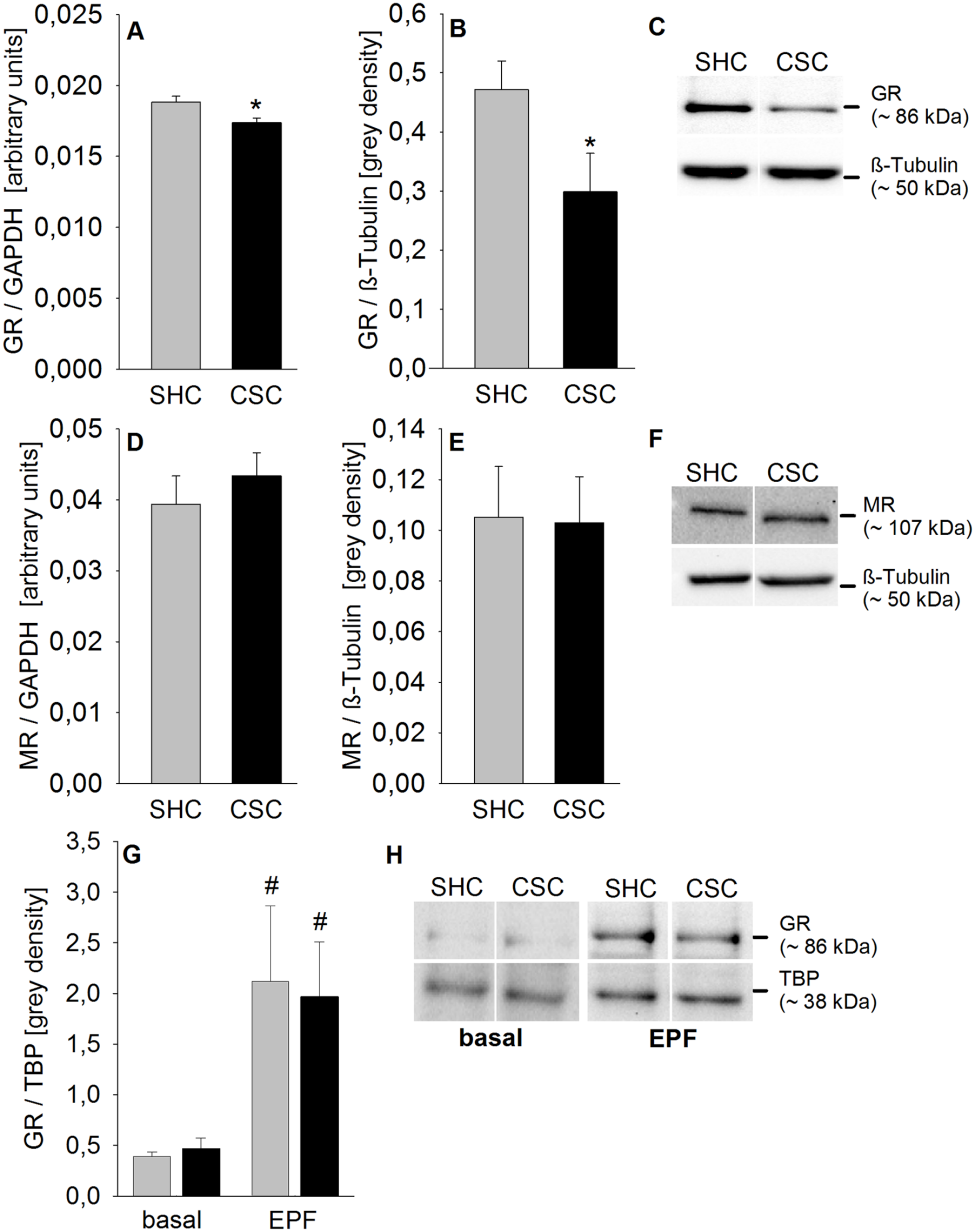
Effects of CSC on hippocampal GR and MR mRNA and cytoplasmic protein expression as well as on nuclear GR protein expression under basal conditions and in response to EPF exposure. Following decapitation on day 20 hippocampi of both SHC and CSC mice were removed. Afterwards, either RNA of SHC (n = 13) and CSC (n = 19) mice was extracted and reversely transcribed into cDNA for quantification of GR and MR mRNA expression [arbitrary units] via qPCR using TaqMan technology normalized to the mRNA expression of the housekeeping gene GAPDH (A/D), or protein was extracted from the hippocampi of SHC (GR: n = 8; MR: n = 6) and CSC (GR: n = 6; MR: n = 6) mice for determination of cytoplasmic GR (B/C) and MR (E/F) protein expression [grey density] normalized to the loading control ß-Tubulin and for determination of basal nuclear GR (G/H) protein expression (SHC: n = 6, CSC: n = 5) normalized to the loading control TATA binding protein (TBP). Another set of SHC and CSC mice was exposed to an elevated platform (EPF) for 5 min on day 20 of CSC. 10 min after termination of EPF exposure, SHC (n = 6) and CSC (n = 7) mice were decapitated, hippocampi were removed and protein was extracted for determination of nuclear GR protein expression [grey density] normalized to the loading control TBP (G/H). Grey bars represent SHC, black bars CSC mice. Data represent the mean + SEM. * represent *P* < 0.05 vs. respective SHC mice; ^#^ represent *P* < 0.05 vs. respective basal. Representative images of bands detected for cytoplasmic GR (~ 86 kDa; C) or MR (~ 107 kDa; F) and respective loading control ß-Tubulin (~ 50 kDa; C/F) are shown for SHC and CSC mice. Representative images of bands detected for nuclear GR (~ 86 kDa; H) and the loading control TBP (~ 36 kDa; B) are shown for SHC and CSC mice under basal and EPF conditions.

Statistical analysis revealed a decrease in GR mRNA (*P* = 0.011; Fig 3A) and cytoplasmic protein (*P* = 0.048; Fig 3B/3C) expression in the hippocampus of CSC compared with SHC mice. In contrast, MR mRNA (Fig 3D) and cytoplasmic protein (Fig 3E/3F) expression were not affected by the CSC exposure. For nuclear GR protein expression in the hippocampus, statistical analysis revealed a significant main effect of factor EPF (F_1,20_ = 10.544; *P* = 0.004). *Post-hoc* Bonferroni pairwise comparisons indicated a significant increase of nuclear GR protein expression in response to 5 min EPF exposure in SHC (*P* = 0.022) and CSC (*P* = 0.047) mice.

### CSC had no effect on FKBP51 protein expression in the hippocampus

**Fig 4:**
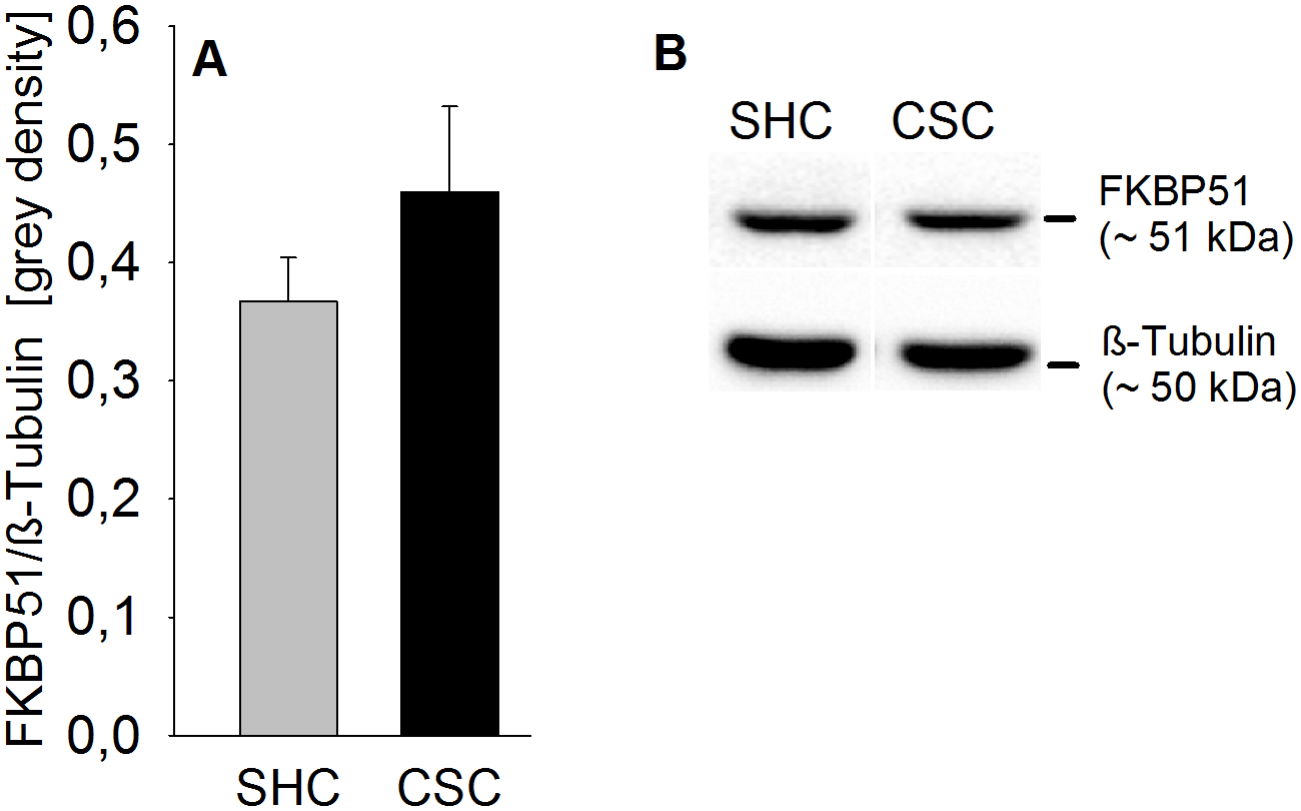
Effects of CSC on FKBP51 protein expression in the hippocampus. Following decapitation on day 20 hippocampi of both SHC (n = 8) and CSC (n = 6) mice were removed and protein was extracted for determination of FKBP51 protein expression [grey density] normalized to the loading control ß-Tubulin (A/B). Grey bars represent SHC, black bars CSC mice. Data represent the mean + SEM. Representative images of bands detected for FKBP51 (~ 51 kDa; B) and the loading control ß-Tubulin (~ 50 kDa; B) are shown for SHC and CSC mice.

Statistical analysis revealed that FKBP51 protein expression in the hippocampus (Fig 4A/4B) was not affected in CSC mice.

### CSC increased CORT-mediated induction of Per1 hnRNA expression in hippocampal cells *in vitro*

**Fig 5:**
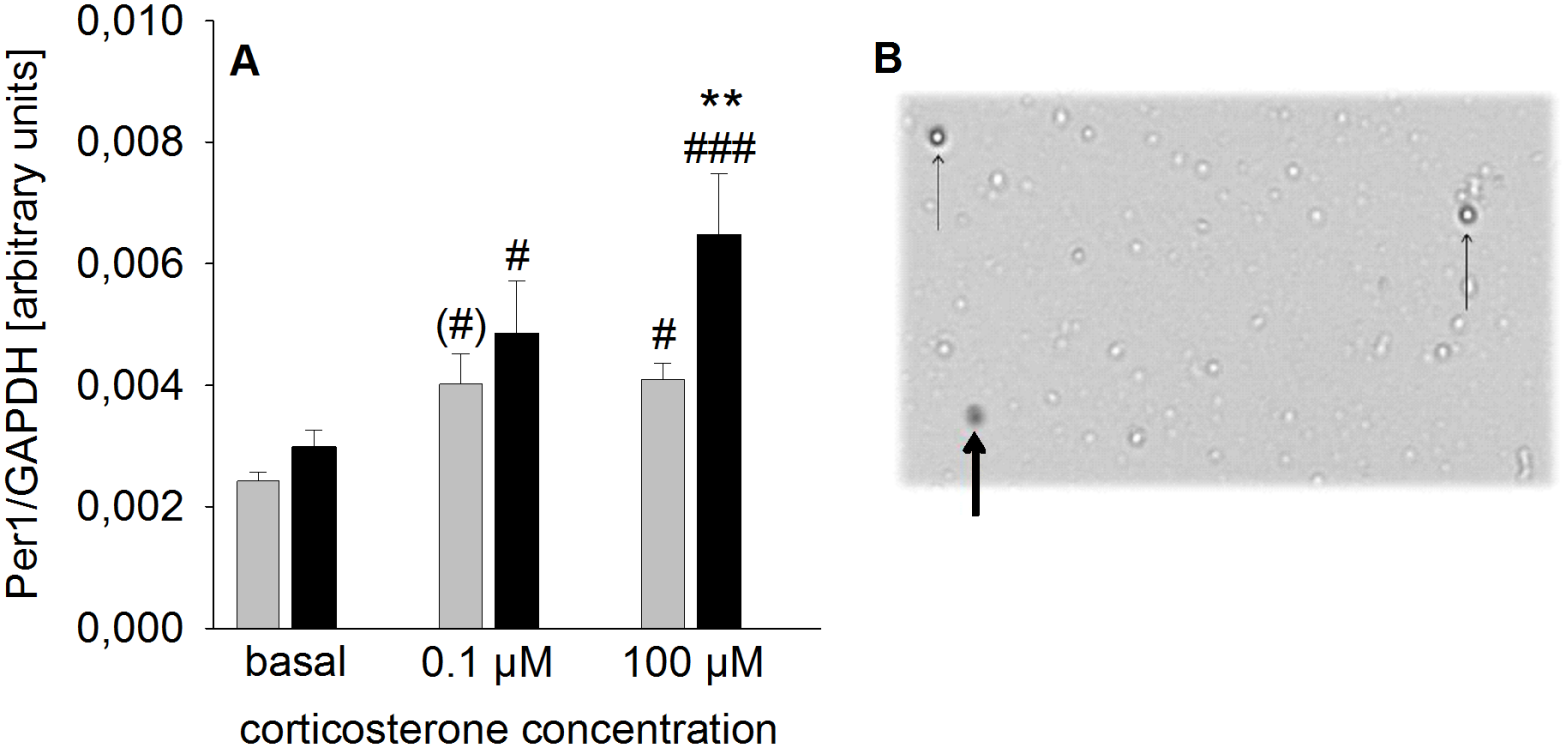
Effects of CSC on CORT-mediated increase in Per1 hnRNA expression in hippocampal cells under *in vitro* conditions. Following decapitation on day 20 hippocampi of SHC and CSC mice were removed and hippocampal cells were isolated. 7 x 10^5^ hippocampal cells were stimulated either with vehicle (basal, SHC and CSC: n = 14), 0.1 μM (SHC and CSC: n = 7) or 100 μM CORT (SHC and CSC: n = 7) for 30 min. Afterwards, RNA of the cells was extracted and reversely transcribed into cDNA for determination of Per1 hnRNA [arbitrary units] via qPCR using TaqMan technology normalized to the mRNA expression of the housekeeping gene GAPDH (A). Grey bars represent SHC, black bars CSC mice. Data represent the mean + SEM. ** represent *P* < 0.01 vs. respective SHC mice; ^#^ represent *P* < 0.05; ^###^ represent *P* < 0.001 vs. respective basal; ^(#)^ represent a trend vs. respective basal. **B** Representative image of the Cell Viability analyzer showing living hippocampal cells (thin black arrow) and a dead cell (bold black arrow).

Statistical analysis revealed a significant main effect of both factor CSC (F_1,50_ = 9.336; *P* = 0.004; Fig 5A) and factor CORT (F_2,50_ = 16.669; *P* < 0.001; Fig 5A) on Per1 hnRNA expression during *in vitro* stimulation of isolated viable (Fig 5B) hippocampal cells with two different CORT concentrations. *Post-hoc* Bonferroni pairwise comparisons indicated a significant increase of Per1 hnRNA expression in response to 100 μM (*P* = 0.049) and a trend towards an increase in response to 0.1 μM (*P* = 0.063) CORT in SHC mice compared with basal conditions. In CSC mice this CORT-induced increase in gene expression was even more pronounced, indicated by significantly elevated Per1 hnRNA expression compared with basal conditions at both 0.1 μM (*P* = 0.023) and 100 μM (*P* < 0.001) CORT. Moreover, Per1 hnRNA expression was higher in hippocampal cells from CSC compared with SHC mice when stimulated with 100 μM CORT (*P* = 0.004).

### CSC affected hippocampal cell viability in response to CORT in a dose-dependent manner

**Fig 6:**
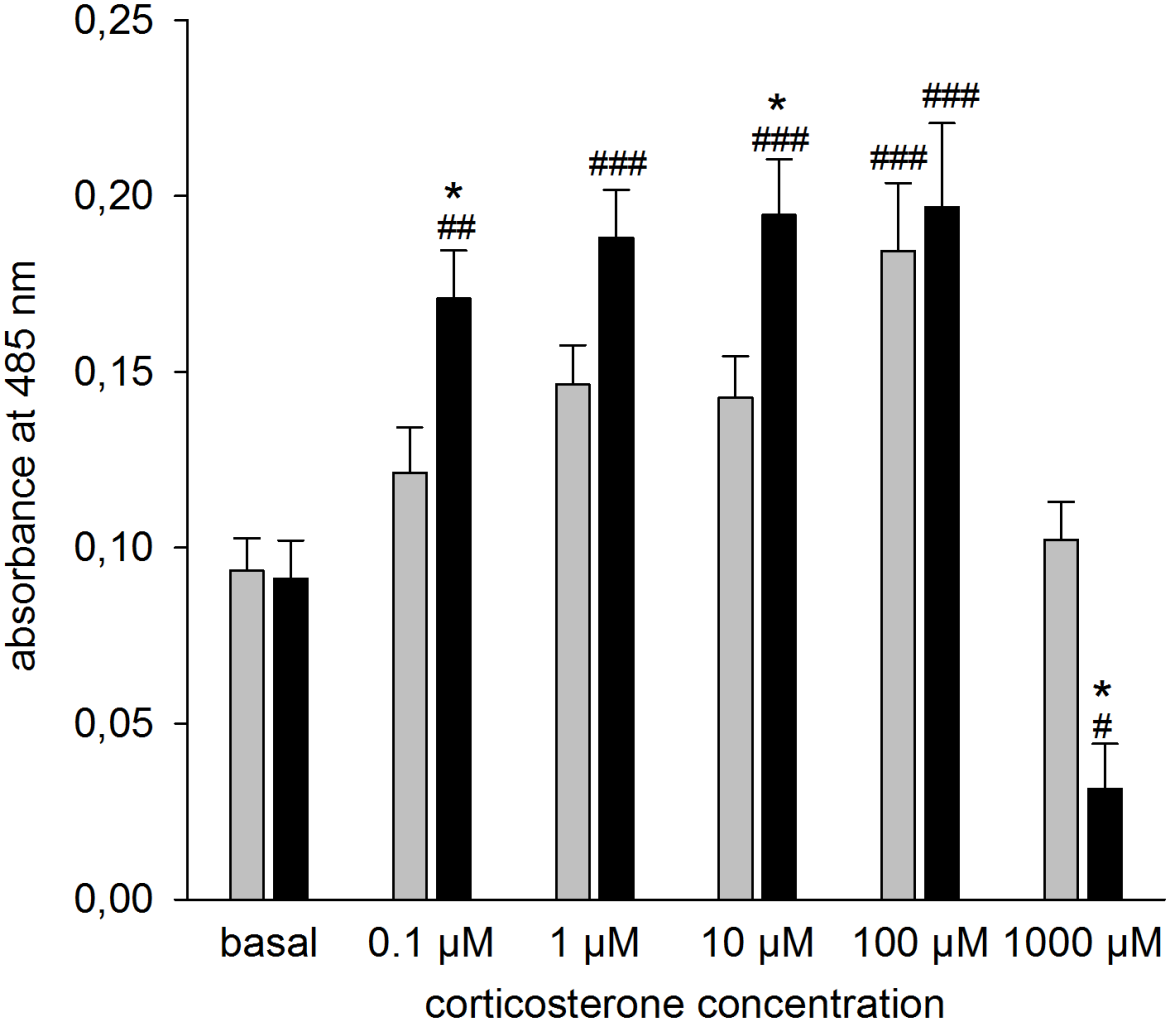
Effects of CSC on CORT-induced changes in hippocampal cell viability *in vitro*. Following decapitation on day 20 hippocampi of SHC and CSC mice were removed and cells were isolated. 2 x 10^5^ hippocampal cells were stimulated with vehicle (basal: SHC: n = 23; CSC: n = 24) or different CORT concentrations (0.1 μM (SHC: n = 11; CSC: n = 9); 1 μM (SHC: n = 11; CSC: n = 9); 10 μM (SHC: n = 11; CSC: n = 9); 100 μM (SHC: n = 12; CSC: n = 15); 1000 μM (SHC: n = 12; CSC: n = 15)) for 24 h and subsequently cell viability [absorbance at 485 nm] was assessed via a commercially available cell viability assay. Grey bars represent SHC, black bars CSC mice. Data represent the mean + SEM. * represent *P* < 0.05 vs. respective SHC mice; ^#^ represent *P* < 0.05; ^##^ represent *P* < 0.01; ^###^ represent *P* < 0.001 vs. respective basal.

Hippocampal cell viability in response to 24 h *in vitro* stimulation with different CORT concentrations was dependent on the interaction of factors CSC and CORT (F_5,149_ = 4.731; *P* < 0.001; Fig 6). *Post-hoc* Bonferroni pairwise comparisons revealed that in CSC mice 0.1 μM, 1 μM, 10 μM and 100 μM CORT and in SHC mice 100 μM CORT increased cell viability compared with respective basal conditions. Following stimulation with 0.1 μM (*P* = 0.039) and 10 μM (*P* = 0.031) CORT, cell viability was higher in CSC compared with respective SHC mice. The highest CORT concentration (1000 μM) decreased cell viability in hippocampal cells of CSC mice compared with basal conditions (*P* = 0.012) and also compared with respective SHC mice (*P* = 0.021).

## Discussion

In the present study we reveal comparable basal as well as acute stress-induced nuclear GR protein expression in the hippocampus of CSC and SHC mice, despite CSC resulted in a pronounced decrease in hippocampal GR mRNA and cytoplasmic protein expression. *In vitro* data even indicate an increased potential of CORT to induce hippocampal Per1 hnRNA expression as well as to affect hippocampal cell viability following CSC exposure, suggesting an, if at all, increased hippocampal GC signaling capacity in CSC versus SHC mice.

The reliability of the CSC paradigm as a chronic psychosocial stress model was confirmed, as CSC versus SHC mice showed an increased absolute adrenal weight, a marker for chronic stress in general [38, 39] and for the CSC paradigm in particular [24, 25, 30]. Moreover, as described previously (for review see [26]) CSC mice show unaffected basal plasma morning CORT concentrations, despite elevated plasma ACTH concentrations. Repeated/ chronic stressor exposure, e.g. repeated immobilization, has been further associated with neuronal atrophy and a decreased number of dendritic branches in the hippocampus [40], as well as a lowering of hippocampal neurogenesis [41]. Magnetic resonance imaging in rats even revealed that repeated immobilization causes hippocampal volume loss [22]. In the present study neither alterations in hippocampus weight nor in the number of isolated hippocampal cells were found following CSC exposure. Given the rather rough nature of these parameters in terms of identifying possible stress-induced structural brain changes, future studies are needed to assess more subtle markers, as for instance dendritic branching.

On a more molecular level, qPCR and western blot analysis of hippocampal tissue revealed a decreased expression of both GR mRNA and cytoplasmic protein in CSC compared with SHC mice, while respective expression levels of the MR were comparable between both groups. The phenomenon of a decreased cytoplasmic GR expression is well known following prolonged GC stimulation, and has been repeatedly described in response to chronic stressor exposure in rats and mice [20, 42, 43]. However, since plasma morning CORT levels in CSC mice are elevated only during the first 24 h after the start of the stress paradigm and return to baseline levels afterwards [24, 34], the sustained effect of CSC on hippocampal GR is unlikely to be solely mediated by changes in plasma CORT concentrations. Of note, in line plasma CORT was also comparable between SHC and CSC mice on day 20 of CSC in the current study. Interestingly, there are studies supporting the idea that high GC concentrations are causative for a first decrease in GR mRNA expression, but not necessarily involved in their sustained down-regulation [42]. Here, other factors, like different classes of neurotransmitters are discussed as possible mediators [44].

Although cytoplasmic GR protein expression was down-regulated following CSC, neither basal nor EPF-induced nuclear GR protein levels were affected, suggesting relative GR ligand sensitivity and/or nuclear translocation rate to be, if at all, increased in CSC versus SHC mice. Notably, plasma CORT concentrations were comparable between CSC (n = 6; 217.40 + 22.97 ng/ml) and SHC (n = 6; 205.15 + 21.78 ng/ml) mice. In terms of the underlying mechanism it is important to mention that GR sensitivity and nuclear translocation capacity are influenced by receptor interplay with coactivators or corepressors and post-transcriptional receptor modifications, like phosphorylation patterns (for review see [45, 46]). Interestingly, phosphorylation pattern changes have been found following chronic social isolation stress in Wistar rats [47], showing unaffected basal and acute stress-induced nuclear GR levels.

Given that the FKBP51 gene contains a GRE and, thus, is under the expressional control of CORT, unaffected cytoplasmic FKBP51 protein levels between CSC and SHC mice further suggest that basal GR DNA affinity and/or transcriptional activity is, at least regarding the FKBP51 gene, not decreased by CSC. In contrast, given that protein expression often contrasts mRNA expression profiles [48] and that *in vitro* CORT-induced *Per1* hnRNA expression was increased following CSC, future studies have to reveal whether *FKPB51* gene transcription and, thus, GR DNA affinity and/or transcriptional activity is even increased following CSC. As FKBP51 in turn inhibits GR affinity and translocation [49], similar FKBP51 protein levels in SHC and CSC mice indicate that increased GR sensitivity and/or translocation, as reported above, is at least not mediated by a reduction in this immunophilin. Together, these *in vivo* findings are in line with studies showing that repeated stressor exposure does not necessarily compromise GR ligand affinity despite decreased hippocampal receptor expression [50]. Recent own data further showed that reduced cytoplasmic GR protein expression in the pituitary of CSC mice was paralleled by an enhanced dexamethasone-induced suppression of HPA axis activity during acute stressor exposure [27].

In line with our *in vivo* data suggesting an if at all increased feedback functionality following CSC exposure despite a decreased GR mRNA and cytoplasmic protein expression, *in vitro* data suggest an increased hippocampal GR signaling capacity and, as a consequence, negative feedback inhibition in CSC versus SHC mice. Here, the potential of CORT to induce hnRNA expression of a GC-responsive gene and to affect hippocampal cell viability in CSC and SHC mice was analysed in isolated hippocampal cells.

Selection of the gene of interest was based on evidence showing that stimulation (for 30 min) of hippocampal slices with 100 nM CORT, a concentration measured following acute forced swim exposure in the hippocampus of rats [51], resulted in an increased Per1 hnRNA transcription [36]. This is in line with evidence demonstrating a GRE in the promoter of the Per1 gene in mice [52]. Interestingly, in the present study isolated hippocampal cells of CSC mice showed a significant increase in Per1 hnRNA expression in response to 0.1 μM (100 nM) CORT, while only a trend towards an increase was found in SHC mice. Moreover, application of a 1000-fold higher CORT dose (100 μM) increased Per1 hnRNA expression in SHC and CSC mice, whereby this effect was significantly more pronounced in CSC compared with SHC mice. Together with our *in vivo* data, these findings strongly implicate that simply measuring a decreased GR mRNA and/or cytoplasmic protein expression does not allow to conclude that the functionality of negative feedback inhibition is compromised.

Prolonged exposure of hippocampal cells to high levels of GC induces neuronal atrophy, an effect that can also be mimicked *in vitro* in primary hippocampal cells [53]. Thus, in the present study, the effects of various CORT concentrations (ranging from 0.1 to 1000 μM) on *in vitro* cell viability of isolated hippocampal cells were assessed in CSC and SHC mice. Interestingly, and in line with the above discussed gene expression data, CORT signaling capacity again seemed to be increased following CSC. Already 0.1, 1 and 10 μM CORT increased cell viability in CSC mice, whereas 100 μM CORT was needed to replicate this effect in SHC mice. Moreover, 1000 μM CORT induced hippocampal cell death and, thus, diminished cell viability in CSC, but not SHC, mice. According to the literature, a CORT-induced impairment of energy metabolism [54, 55], disruption of calcium signaling [56, 57] or increase in glutamate release are possible mechanisms involved in hippocampal cell death (for review see [58]), [59]; similar mechanisms that are involved in brain aging and Alzheimer`s disease (for review see [60]).

Importantly, the fact that GC in the present study were found to regulate *in vitro* viability of hippocampal cells in an inverted U-shaped manner is well in line with the literature. In detail, while low levels of CORT have been shown to increase neuronal excitability via activating mainly the high-affinity MR, high levels of CORT have been shown to decrease neuronal excitability via activating GR in addition to MR (for review see [61–63]). In line with the beneficial role of low CORT doses, adrenalectomy has been shown to cause hippocampal atrophy, an effect restorable by low dose CORT replacement [64]. Therefore, the increased cell viability at low CORT concentrations in CSC but not in SHC mice, might probably be mediated by an increased MR signaling as a result of an altered MR:GR ratio following CSC exposure. Therefore, given that at the CORT concentrations used in both *in vitro* experiments in the present study not only GR but also MR are occupied [7, 65], an involvement of the latter in the increased GC signaling cannot be excluded and has to be assessed in future studies, for instance by employing MR and GR antagonists. A lower cell viability at the highest CORT concentration in CSC but not in SHC mice, is in line with the above discussed increase in overall hippocampal GR signaling capacity following CSC.

Taken together, data of the present study suggest an, if at all, increased GC signaling capacity at the level of the hippocampus in CSC compared with SHC mice, despite a decrease in hippocampal GR mRNA and cytoplasmic protein expression. These data are well in line with the CSC-induced increase in negative feedback function at the level of the pituitary [27] and strongly suggest that the hippocampus, as the pituitary [27], is not involved in mediating the increased plasma ACTH drive found in CSC compared with SHC mice. Furthermore, our findings imply to exhibit caution when extrapolating i) peripheral changes in GC signaling to pituitary/ brain tissues involved in negative feedback inhibition and ii) brain changes in GR mRNA and/ or cytoplasmic protein expression to changes in overall GC signaling/ negative feedback function.
